# Evaluation of drinking patterns and their impact on alcohol-related aggression: a national survey of adolescent behaviours

**DOI:** 10.1186/1471-2458-13-950

**Published:** 2013-10-10

**Authors:** Valeria Siciliano, Lorena Mezzasalma, Valentina Lorenzoni, Stefania Pieroni, Sabrina Molinaro

**Affiliations:** 1 Institute of Clinical Physiology, National Research Council (IFC-CNR), Via Moruzzi, 1, 56124 Pisa, Italy

**Keywords:** Drinking patterns, Alcohol-related aggression, Adolescents, ESPAD

## Abstract

**Background:**

Although there have been a wide range of epidemiological studies examining the impact of patterns of alcohol consumption among adolescents, there remains considerable variability in both defining these patterns and the ability to comprehensively evaluate their relationship to behavioural patterns. This study explores a new procedure for defining and evaluating drinking patterns and integrating well-established indicators. The composite measure is then used to estimate the impact of these patterns on alcohol-related aggressive behaviour among Italian adolescents.

**Methods:**

Data were collected as part of the 2011 European School Survey Project on Alcohol and other Drugs (ESPAD). A national sample of 14,199 students aged 15–19 years was collected using an anonymous, self-administered questionnaire completed in a classroom setting. Drinking patterns were established using principal component analysis. Alcohol-related aggression was analysed as to its relationship to patterns of drinking, behaviour of friends towards alcohol use, substance use/abuse, school performance, family relationships and leisure activities.

**Results:**

Several specific drinking patterns were identified: “Drinking to Excess” (DE), “Drinking with Intoxication” (DI) and “Drinking but Not to Excess” (DNE). A higher percentage of males were involved in alcohol-related aggression compared with females. In males, the DE and DI patterns significantly increased the likelihood of alcohol-related aggression, whereas the DNE pattern was negatively associated. Similar results were found in females, although the DI pattern was not significantly associated with alcohol-related aggression. Overall, cigarette smoking, illegal drug use, truancy, limited parental monitoring, frequent evenings spent outside of the home and peer influence associated strongly with alcohol-related aggression.

**Conclusions:**

Our findings suggest that drinking patterns, as uniquely monitored with an integrated metric, can: 1) explain drinking habits better than commonly used indicators of alcohol use and 2) provide a better understanding of behavioural risks such as alcohol-related aggression. Environmental background also appears to strongly associate with this type of aggressive behaviour.

## Background

Alcohol consumption among adolescents is a common concern that is growing in most countries and, notably, hazardous and harmful drinking patterns seem to be on the rise [1,2]. There is extensive evidence that alcohol and physical aggression are associated. It has been shown that adolescents who display violent behaviour are more likely to exhibit problematic alcohol consumption than other adolescents [3,4], excessive alcohol use or drunkenness [5]. Similarly, it has been demonstrated that adolescents who misuse alcohol have higher rates of violent behaviours [6-8]. According to Fagan [9], alcohol consumption provides a “provocative context” for violence, rather than a direct cause, whereas another study proposes alcohol consumption as a moderating variable with a conditioning and reinforcing role in explaining aggressive behaviour [10]. Results from a longitudinal study [11], focusing on the relationships between alcohol misuse, antisocial behaviour and alcohol-related problems at particular ages, strongly support the reciprocal hypothesis. Alcohol misuse and antisocial behaviour establish a “feedback loop” in a joint-effects model (cross-sectional data on 15 years old drinkers), whereas the susceptibility hypothesis (i.e. people with susceptibility to, or on a trajectory towards antisocial behaviour, use alcohol to a greater extent than those who are less susceptible) is prevalent in the shorter term model. Similarly, results from a study on alcohol and violence [12] suggest that alcohol has a “magnifying” effect, amplifying underlying aggressive tendencies. Even experimental studies support a strong relationship between acute alcohol consumption and aggressive behaviour, confirming the idea that acute alcohol consumption facilitates or increases the expression of aggressive behaviour (for a review see Giancola [13]).

In the body of research on the relationship between alcohol consumption and aggression, aggressive behaviours have been investigated in many respects. Our study deals with alcohol-related aggression, defined as the co-occurrence of drinking and physical fighting within a single episode [14]. In this instance, questions remain concerning which alcohol consumption indicators are most suitable to measure the impact on aggressive behaviours. Although the “average amount of alcohol consumed” is consistent with a range of physical and social consequences, a growing number of behavioural studies provide evidence that not only quantity but also patterns of drinking are measures that relate to drinking outcomes [1,15,16] and, notably, to alcohol-violence association [4]. Besides frequency of use, a basic parameter indicating the regularity of drinking, one of the most studied characteristics is binge drinking (typically defined as consuming five or more drinks on a single occasion), which exhibits high prevalence among youth [1,17]. Other studies, focused on alcohol’s negative effects, report that the extent of drunkenness (or perceived intoxication) rather than total volume of alcohol consumed relates to acute consequences such as various types of aggression and violence [4,18-20]. All the indicators, above, of alcohol use, if combined, can capture the diversity of drinking customs. However, a difficulty arises when they are used concurrently in multivariate analyses, due to multicollinearity that may result from the high functional correlation among them. To overcome this difficulty, we propose a different characterization of drinking patterns that constructs a composite metric that combines standard alcohol use parameters such as frequency of consumption, frequency of binge drinking, and frequency of perceived intoxication. This new analytical approach could provide more detailed information on the relationship between alcohol consumption and aggression. Moreover, since it has been demonstrated [21,22] that preferences for alcoholic beverage may reflect different attitudes towards alcohol consumption, such preferences were also taken into account.

The connection between alcohol consumption and aggressive behaviour operates at multiple levels and is the result of a dynamic interplay among personal and socio-environmental systems [23]. This comprehensive approach, incorporating concepts derived from problem-behaviour theory [24], offers a theoretical framework for better understanding underage alcohol use. A broad array of factors potentially affecting alcohol-related aggression in adolescents has been identified in previous research. For this reason, a number of individual, environmental and behavioural variables that may be either “protective” or a “risk” factor for adolescent behaviour problems were also evaluated. Both violent behaviour and alcohol drinking undergo significant changes during adolescence and age and gender can characterize both behaviours. The same applies to substance use and leisure time activity. Another set of factors, whose influence has been repeatedly assessed, involves the socio-environmental system. In this context, family function vs dysfunction, as well as school performance, and peer influence are well-documented confounders that can impact involvement in alcohol-related aggression [13,18,23].

Thus, the purpose of the present study was threefold: a) to test the ability of observed drinking patterns for association with alcohol-related aggression, b) to determine if drinking patterns, as evaluated in this study, add value to common indicators of alcohol use, and c) to verify the role of a number of factors as mediators between drinking patterns and alcohol-related aggression.

## Methods

A full description of sampling and data collection procedures has been reported in the 2011 European School Survey Project on Alcohol and Drugs (ESPAD) Report [25]. Briefly, standardized data collection was performed using an anonymous self-administered questionnaire completed on a voluntary basis in the classroom setting. The authorization of the school head to fill in the Italian ESPAD questionnaire by the students was required. The survey was included in the Scholastic Plan for Education (Decree of the President of the Italian Republic n.275/1999, Art. 8), edited, decided and approved by Collegial Bodies, including teachers, parents and students (Legislative Decree n.297/1994). All analyses in the present study are based on data from the 2011 Italian ESPAD survey, provided by the Institute of Clinical Physiology, National Research Council (IFC-CNR). Data can be obtained with appropriate permission. Of the sampled schools, 89% participated in the survey. The target population was comprised of Italian high-school students aged 15–19 years. Less than 0,5% of the students refused to participate in the study.

### Participants

The sample included respondents who reported consuming alcohol at least once during the previous year. Experiences of alcohol-related aggression also refers to the same period. Of 18,427 participants in the 2011 ESPAD survey, 15,026 reported consuming alcohol in the last year. Only those students that consistently answered questions about chosen indicators of alcohol use were included in the analysis (n = 14,199 students; 7290 males and 6909 females; age 17.2 ± 1.4 years [mean ± SD]).

### Alcohol use indicators

Indicators of alcohol consumption were assessed for the month prior to the survey (i.e. among current drinkers). Three questions from the 2011 ESPAD core questionnaire were used as screens:

a) “During the last 30 days, on how many occasions have you had any alcohol beverage to drink?”

b) “Think back over the last 30 days. How many times have you had five or more drinks on one occasion?”

c) “During the last 30 days, on how many occasions (if any) have you been intoxicated from drinking beverages, for example staggered when walking, not being able to speak properly, throwing up or not remembering what happened?”

Questions a) and c) had 7 response categories: “0, 1–2, 3–5, 6–9, 10–19, 20–39 and 40+ occasions”, while question b) had 6 response categories: “none, 1, 2, 3–5, 6–9, 10+ times”.

Alcoholic beverage preferences and their frequency of use were also considered using the question “Think back over the last 30 days. On how many occasions have you had any of the following to drink?” Possible choices were beer, alcopops, wine, and spirits (beverages with high alcoholic content) with response categories “0, 1–2, 3–5, 6–9, 10–19, 20–39, 40+ occasions”.

### Alcohol-related aggression

Alcohol-related aggression was evaluated on the basis of the following question:

“Because of your own alcohol use, how often during the last 12 months have you experienced physical fighting?”

The response categories were “0, 1–2, 3–5, 6–9, 10–19, 20–39 and 40+ occasions”.

Due to the low number of observations reported in the upper ranges, the response was dichotomized (0 vs ≥1 occasion).

“Experienced physical fighting” is intended as direct involvement in a fight. We use the term “aggression” in place of “physical fighting” in the current text.

### Other variables

Some other variables potentially affecting adolescent behaviour were also considered. These parameters were grouped as follows:

a) family: parental monitoring (parents know where students spend Saturday nights always/quite often vs sometimes/usually don’t know); family structure (living with both parents vs one parent/others);

b) substance use: use of substances (cannabis, other illegal drugs) at least once during the last year vs none; having smoked cigarettes daily during the last month vs less than one cigarette per day (including not at all);

c) school: having missed school, without a valid reason, for 3 days during the last month vs less than 3 days; having obtained high marks in the last term vs low/medium marks;

d) frequent (almost daily) vs infrequent (at least once a week or less) leisure time activity: sports practice, going out in the evening (to a disco, café, party etc.), slot machines gambling (actual betting of money);

e) friends’ behaviour with alcohol: categorized as non-drinkers, regular drinkers but few get drunk, regular drinkers and most get drunk.

### Statistical analysis

Principal component analysis (PCA) [26] was applied to the three indicators of alcohol consumption, expressed as frequencies, to obtain three independent factors representing different drinking patterns and used simultaneously in the regression model. PCA extracts a set of principal components (or factors) obtained as a linear combination of the original indicators. No rotation procedures were required to facilitate the interpretation of the factors. The contribution of each indicator is the loading (signed) derived from the analysis. A positive loading means that higher levels of an indicator are associated with higher levels of that factor and a negative loading means that lower levels of an indicator are associated with higher levels of that factor. Each principal component represents a certain amount of total variance in the data: by using all components the total amount of variance is conserved. The components obtained were interpreted in terms of different alcohol drinking patterns.

Each pattern can be treated as a numerical variable similar to an assessment scale, with a minimum and a maximum value. In each pattern, an increase of one unit must be interpreted in terms of the composite indicators and their specific contributions. For example, a pattern would result from a linear combination of the three indicators with positive loadings (e.g. drinking pattern = loading1*use + loading2*intoxication + loading3*binge drinking), characterized by a scale that increases with frequency of alcohol use or binge drinking or intoxication, separately, or in combination. In this pattern, the minimum drinking pattern value is equal to zero and means no alcohol use in the last month (and therefore no intoxication or binge drinking) and the maximum value is equal to the highest frequency of intoxication and binge drinking (and, consequently, also by high frequency of alcohol use).

Pearson’s correlation was used to explore the relationship among the three indicators and between drinking patterns and frequency of use of specific alcoholic beverages (beer, wine, alcopops and spirits). Logistic regression analysis was performed to verify the association between aggressive behaviour and alcohol consumption, evaluated both as individual indicators and as drinking patterns. Individual indicators and drinking patterns were treated as continuous (ordered categorical) variables. Three models were evaluated: univariate logistic regression using indicators representing alcohol use (Model 1), multivariate logistic regression using indicators indicating alcohol use (Model 2) and multivariate logistic regression using drinking patterns (Model 3). Results are reported using beta coefficients and standard errors, odds ratios (OR) and 95% confidence interval (CI). Univariate and multivariate logistic regression models (Model 1 and 2, respectively) were used to control for potential confounding effects. Alcohol indicators were tested for confounding effects by jointly introducing them into the model and examining beta coefficients: a change in beta coefficient greater than 10% was considered to be a source of confounding. In Model 2, multicollinearity among independent indicators of alcohol use was also evaluated using the variance inflation factor (VIF): a VIF that exceeded 5 was taken as an indication of multicollinearity [27].

All other personal and behavioural variables had been previously tested using univariate analysis and those that appeared statistically significant (p < 0.05) were included in the multivariate regression model (adjusted odds ratios) along with drinking patterns.

All the analyses were performed separately against gender. Statistical significance was set at p < 0.05 (two-tailed). All the analyses were performed using Stata software, version 10.1.

## Results

### Descriptive statistics

Overall, the majority of students who have consumed alcohol during the last year were also current drinkers: in fact, 82% of them (78% females and 86% males, respectively) consumed alcohol at least once in the last month. Among them, alcohol use without binge drinking or perceived intoxication was a common habit (39%); binge drinking without any experience of perceived intoxication was also significant (27%), whereas perceived intoxication alone occurred infrequently (2%). Finally, 14% of students reported at least one experience of binge drinking and at least one of perceived intoxication. Significant (p < 0.05) correlations were found among the three indicators: for alcohol use and binge drinking r = 0.57, for alcohol use and perceived intoxication r = 0.40, for binge drinking and perceived intoxication r = 0.47. Regarding alcohol-related aggression, about 12% of students have been involved, predominantly males (18%; females, 6%), but only 3.7% of adolescents reported involvement more than twice.

### Drinking patterns

Drinking patterns were calculated on the basis of current alcohol consumption (i.e. in the month prior to the survey) among adolescents who had consumed alcohol at least once in the last year. There was a portion of adolescents (18%) who did not use alcohol in the month prior to the survey. Drinking patterns identified by PCA were defined as 1) Drinking to Excess (DE); 2) Drinking with Intoxication (DI); and, 3) Drinking but Not to Excess (DNE). Table 1 summarizes the PCA results. DE pattern accounted for 65% of the total variance and was characterized by a positive correlation with the frequency of all individual indicators (0.582*alcohol use + 0.540*intoxication + 0.608*binge drinking) and is interpreted as the pattern of those who 1) drink frequently, 2) report perceived intoxication and 3) do binge drinking. When using a composite numerical variable, the minimum value was zero (all indicators were equal to zero, i.e. no alcohol was consumed during the last month) and the maximum value was 10 (the highest frequency of both intoxication and binge drinking). Thus, an increase of one unit in DE pattern indicated an increase in frequency of excessive alcohol use. DE pattern showed that experience of excessive drinking alcohol among adolescents is both related to binge drinking and to experiencing intoxication. DI pattern exhibited 21% variance and also a negative correlation with frequency of alcohol consumption, a positive high correlation with perceived intoxication and a small negative correlation with frequency of binge drinking (−0.538*alcohol use + 0.816*intoxication − 0.210*binge drinking) and it is interpreted as the pattern of those who 1) drink infrequently, 2) report perceived intoxication, but 3) do not binge drink. The corresponding minimum composite value was −4 (the highest frequency being binge drinking) and the maximum value was 2 (the highest frequency being intoxication). An increase of one unit in DI pattern indicated an increase in frequency of perceived intoxication during every drinking experience and a decrease in frequency of binge drinking. DI pattern showed that perceived intoxication among adolescents was not necessarily linked to binge drinking, and there was a proportion of them who experienced intoxication even if they drank infrequently. DNE pattern exhibited a variance of 14% and a positive correlation with frequency of alcohol consumption, a positive, but low correlation with frequency of perceived intoxication and a negative high correlation with frequency of binge drinking (0.610*alcohol use + 0.205*intoxication − 0.766*binge drinking) and it is interpreted as the pattern of those who 1) drink frequently, 2) do not report perceived intoxication, and 3) infrequently binge drink. The corresponding minimum ordinal value was −1 (the highest frequency being binge drinking) and the maximum value was 5 (the highest frequency being alcohol use). An increase of one unit in DNE indicated an increase in frequency of alcohol consumption without binging. DNE pattern showed that there was a portion of adolescents who experienced moderate alcohol use, drinking frequently but without experiencing binge drinking or perceived intoxication.

**Table 1:** Factor loadings, eigenvalues and explained variance of principal components

	**I component**	**II component**	**III component**
	**(Drinking to excess)**	**(Drinking with intoxication)**	**(Drinking but not to excess)**
Frequency of alcohol use	0.582	−0.538	0.610
Frequency of perceived intoxication	0.540	0.816	0.205
Frequency of binge drinking	0.608	−0.210	−0.766
Eigenvalue	1.97	0.61	0.42
Explained variance	65%	21%	14%

**Figure 1:**
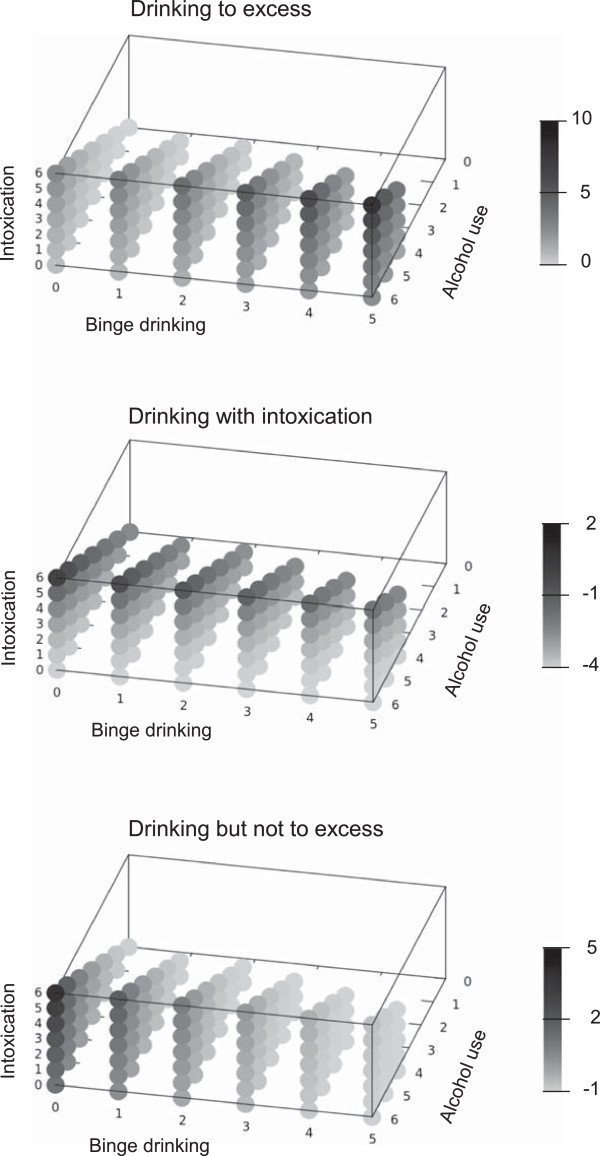
**Three-dimensional representation of the drinking patterns.** “Drinking to excess” **(upper panel)**, “Drinking with intoxication” **(central panel)** and “Drinking but not to excess” **(lower panel)** obtained through principal component analysis. Each point represents the value of the pattern corresponding to the combination of the three indicators (perceived intoxication, binge drinking, and alcohol use). Shade of grey varies from light to dark grey (from lowest to highest value).

Figure 1 shows a three-dimensional representation of the aforementioned drinking patterns: points represent all the theoretical values that the specific pattern could assume in correspondence of the combination of frequencies of the three selected indicators. The points are presented in shades of grey, from light grey at the lowest value to dark grey at the highest. As shown, ED pattern had higher values at increasing frequencies of all indicators, DI pattern had higher values at increasing frequency of both alcohol use and intoxication, DNE pattern had higher values in correspondence with higher frequency of alcohol use and lower frequency of binge drinking.

### Correlation between drinking patterns and alcoholic beverages

Pearson correlation between the DE pattern and frequency of the specific alcoholic preferences resulted in significant correlations with all beverages (r = 0.52 for wine, r = 0.62 for beer, r = 0.56 for alcopops, and r = 0.68 for spirits), whereas significant negative correlations were reported for the DI pattern (r = − 0.41 for wine, r = − 0.52 for beer, r = − 0.44 for alcopops, and r = − 0.50 for spirits; negative values are due to infrequent use of alcohol). For DNE pattern, weak significant positive correlations were found with all beverages (r = 0.06 for spirits, r = 0.08 for alcopops, r = 0.10 for wine, and r = 0.10 for beer).

### Association between alcohol consumption and alcohol-related aggression

The association of individual alcohol use indicators and drinking patterns with alcohol-related aggression was tested using logistic regression analysis. Table 2 shows results from several models. First, individual alcohol indicators (frequencies of alcohol use, binge drinking, and perceived intoxication) were analysed in a univariate model (Model 1): the higher the frequency of the three indicators, the higher the likelihood of being involved in alcohol-related aggression and in both genders. Second, these alcohol use indicators were analysed using a multivariate model (Model 2): although, as expected, a positive association with alcohol-related aggression was found for all the indicators, changes in beta coefficients of more than 30% denoted a substantial confounding effect. Mean value of VIF was greater than 5 (males: 5.1, females: 5.3), with the highest value for frequency of alcohol consumption (males: 5.7, females: 5.5). Finally, in Model 3 drinking patterns, only, were examined: in both genders, all were significantly associated with alcohol-related aggression, but while DE and DI patterns correlated positively, DNE pattern had a negative correlation.

**Table 2:** Association of alcohol consumption indicators and drinking patterns with alcohol-related aggression using logistic models

	**Alcohol-related aggression**			
	**Males**		**Females**	
	**β (SE)**	**OR (95% CI)**	**β (SE)**	**OR (95% CI)**
Model 1^a^				
Alcohol indicators				
Frequency of alcohol use	0.400 (0.019)	1.49 (1.44–1.55) ***	0.504 (0.032)	1.66 (1.56–1.76) ***
Frequency of binge drinking	0.564 (0.020)	1.76 (1.69–1.83) ***	0.691 (0.034)	2.00 (1.87–2.13) ***
Frequency of perceived intoxication	0.932 (0.044)	2.54 (2.33–2.77) ***	1.129 (0.068)	3.09 (2.71–3.53) ***
Model 2^b^				
Alcohol indicators				
Frequency of alcohol use	0.096 (0.024)	1.10 (1.05–1.15) ***	0.149 (0.043)	1.16 (1.07–1.26) ***
Frequency of binge drinking	0.398 (0.025)	1.49 (1.42–1.56) ***	0.480 (0.044)	1.62 (1.48–1.76) ***
Frequency of perceived intoxication	0.486 (0.045)	1.63 (1.49–1.78) ***	0.505 (0.075)	1.66 (1.43–1.92) ***
Model 3^c^				
Drinking patterns				
Drinking to excess	0.559 (0.023)	1.75 (1.67–1.83) ***	0.650 (0.036)	1.92 (1.79-2.05) ***
Drinking with intoxication	0.259 (0.042)	1.30 (1.19–1.41) ***	0.230 (0.072)	1.26 (1.09–1.44) ***
Drinking but not to excess	−0.144 (0.031)	0.87 (0.81–0.92)***	−0.171 (0.054)	0.84 (0.76–0.94) **

β: beta coefficient, SE: standard error, OR: odds ratios, CI: confidence interval, ** p < 0.01, *** p < 0.001.

^a^ univariate model, ^b^ and ^c^ multivariate models.

Table 3 reports results from logistic regression between alcohol-related aggression and drinking patterns, controlling for the other variables. No changes in drinking patterns’ association were observed in males, whereas in females DI pattern was no longer significantly associated with alcohol-related aggression. Age was negatively associated with alcohol-related aggression only for males. Investigating the influence of the drinking habits of peers, having many friends who become intoxicated was more likely observed in male adolescents who exhibited alcohol-related aggression.

**Table 3:** Multivariate logistic analysis of alcohol consumption patterns and other correlates of alcohol-related aggression

	**Alcohol-related aggression**	
	**Males**	**Females**
	**adj OR (95% CI)**	**adj OR (95% CI)**
Drinking patterns		
Drinking to excess	1.41 (1.34–1.49) ***	1.62 (1.49–1.77) ***
Drinking with intoxication	1.20 (1.09–1.31) ***	1.17 (1.00–1.37)
Drinking but not to excess	0.88 (0.82–0.94) ***	0.86 (0.77–0.97) *
Age	0.90 (0.85–0.96) ***	…§
Friends’ behaviour with alcohol		
Regular drinkers but few get drunk	1.24 (0.80–1.92)	…
Regular drinkers and most get drunk	2.06 (1.32–3.21) ***	…
Substance use		
Cannabis in the last year	1.68 (1.40–2.01) ***	1.86 (1.41–2.45) ***
Other illegal substances	2.32 (1.79–3.01) ***	2.96 (2.04–4.28) ***
Daily use of cigarettes in the last month	1.51 (1.26–1.82) ***	…
School		
Truancy (≥3 days in the last month)	1.43 (1.18–1.72) ***	1.51 (1.11–2.04) **
Scholastic achievement in the last term	0.82 (0.70–0.96) *	0.76 (0.60–0.98) *
Family		
Parental monitoring (frequent or always)	0.68 (0.57–0.80) ***	…
Leisure time		
Sports activities (almost daily)	1.49 (1.27–1.74) ***	…
Going out in the evening (almost daily)	1.44 (1.22–1.70) ***	1.37 (1.05–1.80) *

adj OR: adjusted odds ratios, CI: confidence interval, * p < 0.05, ** p < 0.01, *** p < 0.001.

§ Ellipses indicate that the variable was excluded from the multivariate model because of p ≥ 0.05.

Overall, illegal drug use, truancy, and frequent evenings spent outside of the home were habits strongly associated with alcohol-related aggression and without distinction of gender. Cigarette smoking showed a strong association but only for males. Other factors such as achieving high marks at school and high level of parental monitoring (the last only in males, in females this variable was not included in the multivariate analysis) were negatively associated with alcohol-related aggression, whereas frequent participation in sports, evaluated only in males, was more likely associated with alcohol-related aggression.

## Discussion

Regardless of the minimum legal drinking age (16 years old in Italy), the experience of drinking alcohol is widespread among young people in Italy. Our findings have shown that alcohol consumption was higher in male students (86%) and that, for both genders, alcohol use without major consequences predominated (39%) even if binge drinking was observed as a widespread pattern (27%). It can be also observed that binge drinking and perceived intoxication occurred jointly (14%), supporting the idea of identifying and using patterns of drinking – resulting in composite relationships – rather than evaluating alcohol habits using a single indicator or by type/quantity of beverage consumed. In addition, although the ESPAD questionnaire clearly defines a “drink” (i.e. a glass/bottle/can of beer (25–33 cl), a glass/bottle can of cider (25–33 cl), a bottle of alcopops (27 cl), a glass of wine (10–12.5 cl), a glass of spirits (4 cl) or a mixed alcoholic beverage), it remains quite difficult to estimate the actual amount of ethanol consumed as well as the individual sensitivity to any specific alcohol volume.

For this reason, using PCA results, we have examined the impact of three styles of alcohol consumption on alcohol-related aggression: 1. alcohol consumption leading to DE pattern, the greatest risk; 2. consuming alcohol infrequently but leading to perceived intoxication (DI pattern), popular behaviour among adolescents in the so called “dry” countries, but now also increasing in Italy; 3. drinking but not to excess (DNE pattern), a “moderate” approach that is poorly studied.

Regarding alcoholic beverage preferences, the DE pattern appeared to correlate with consumption frequency of spirits more than other alcoholic beverages, while DI negatively correlated with consumption frequency of beer to a greater extent. Lastly, the DNE pattern correlated poorly with all beverages. It would be important to explore this in future studies using the ESPAD data, particularly comparing different drinking cultures to assess whether drinking patterns are connected to new drinking habits (e.g. concurrent consumption of alcoholic beverages and energy drinks).

In investigating the relationship between aggressive behaviours and alcohol use, some studies have considered single parameters to assess drinking [23,28], whereas other studies have evaluated the interaction of several parameters to characterize different classes of drinkers [29] or have utilized a single drinking pattern score obtained by combining several indicators [30]. The analysis of drinking patterns is a key factor in alcohol-related aggression as it draws information which otherwise cannot be inferred. In our study, the main advantage of this approach was to highlight a drinking pattern that has so far been little studied: in fact, our study not only confirms what is already known, i.e., excessive drinking is associated with an increased likelihood of alcohol-related aggression but it has also identified a negative association with moderate drinking: in other words, moderate drinking significantly decreases the likelihood of being involved in alcohol-related aggression, a finding confirmed in both genders. It should be further investigated as to whether this is due to a “protective” role of this drinking pattern or to a more general moderate (without excess) behaviour. Actually, little is known about young people consuming alcohol at “low-risk” levels: only recently, research has addressed this issue in an attempt to establish drinking guidelines for youth [31]. For this reason, the DNE pattern deserves greater attention in future studies because it is a drinking style adopted widely among young Italian drinkers and a thorough understanding of this pattern may provide additional perspectives on other behaviours. In addition, DI pattern also merits comment: although less strongly associated with alcohol-related aggression compared to DE pattern, it provides an insight into the risky behaviour of those who drink infrequently without experiencing binge drinking, reflecting the fact that alcohol-related aggression is not exclusively related to frequent or compulsive drinking.

Many other factors enter into the relationship between aggressive behaviour and alcohol use among adolescents. Some gender differences were detected by the logistic regression model: in fact, while in males the relationship between alcohol-related aggression and all the three drinking patterns was not modified by the potentially confounding variables, in females the association between alcohol-related aggression and DI pattern was no longer present. This finding suggests that, in females, socio-environmental characteristics underlying alcohol-related aggression and DI pattern are similar and therefore responsible for the association. Alcohol-related aggression varied by age only in males, demonstrating that involvement in alcohol-related aggression was more likely in younger males and that this behaviour gradually changes through the teenage years. The effect of age appears therefore relevant, especially in males, as shown also in previous studies [18,28]. Furthermore, the drinking behaviour of peers has often been considered influential. Compared to other studies that have evaluated only the number of peers who drink, in our study we have considered different friends’ habits: non-drinkers, regular drinkers but few get drunk, regular drinkers and most get drunk: from our results, associating with friends who drink alcohol doesn’t appear influential as long as they do not consume alcohol in excess. As observed by others [18], the gender-specific analysis reveals significant differences and underlines the differential impact, for males and females, of drinking patterns and of the other factors that play a role in alcohol-related aggression. Other correlates were equally relevant for both genders. As already shown [32], the use of illegal drugs resulted as always positively associated with alcohol-related aggression, indicating a tendency to concurrent problem behaviours. In addition, truancy and simply spending many evenings outside the home environment represent attitudes that can contribute to problematic behaviours, and, as found in our study, to alcohol-related aggression involvement. Overall, from the outcomes of the analysis, it can be argued that alcohol-related aggression in young people is more commonly associated not only with drinking to excess, but also with a number of features that express discomfort, converging towards an overall risk-taking behaviour.

Some limitations of the study should be mentioned. First, data were derived from a school-based sample of adolescents, thus excluding school dropouts, and were self-reported. Second, the definition of alcohol-related aggression was based on a question that asked participants if they had experienced physical fighting “*because of your own alcohol use*”. In order to answer to this question, the participants must attribute their fighting behaviour to their alcohol use. Since the question specifically asks participants only about fighting behaviours that were attributed to drinking, our analyses may underestimate the number of all fighting and drinking that co-occur, since a portion of these occurrences may not have been attributed to the drinking. Third, conclusions on the causal relationships cannot be drawn as the data were cross-sectional. Moreover, we recognize the lack of other important indicators such as the volume of drinking or the drinking context that could provide more comprehensive information regarding alcohol-related aggression.

## Conclusions

Our results suggest that alcohol consumption, alcohol-related aggression and their relationship are the result of a more complex system in which many other factors play important roles, leading to overall risk-taking behaviours. Therefore, for long-term impact, efforts to reduce aggressive behaviour in youths and policies aimed at curbing alcohol use need to adopt a “whole system” approach that should include both regulatory interventions and concomitant strategies for reducing the negative consequences of problems once they have emerged. With specific regards to alcohol consumption, interventions involving education and returning to Mediterranean cultural traditions (drinking in small amounts, preferably during meals), could possibly encourage a more responsible approach to alcohol consumption and a greater awareness of the consequences of excessive drinking.

## Competing interests

The authors declare that they have no financial competing interests.

## Authors’ contributions

SM obtained the funding for the study. VS and SM developed the study design. LM managed the literature searches and summaries of previous related work. VS and VL undertook the statistical analysis. VS, SM, SP, and LM interpreted the data. LM and VS wrote the initial draft of the manuscript. All authors contributed to and have approved the final manuscript.

## Pre-publication history

The pre-publication history for this paper can be accessed here:

http://www.biomedcentral.com/1471-2458/13/950/prepub
